# Iatrogenic Hypotension Induced by the Improper Treatment of Normal Grief

**DOI:** 10.1155/2021/6661943

**Published:** 2021-04-13

**Authors:** Kyle E. Robinson

**Affiliations:** Mayo Clinic, Rochester, MN, USA

## Abstract

We report the case of a 28-year-old male in rural Madagascar with iatrogenic hypotension induced by improper treatment of a normal grief response. The man lost both of his children in the spring of 2019 during a measles outbreak that infected at minimum 152,000 individuals on the island. After developing symptoms of chest pain, intermittent tachycardia, and widespread pain when he would think of his children in the weeks following their loss, he was prescribed gabapentin, lisinopril, and metoprolol by a general practice nurse. He subsequently developed dizziness, lightheadedness, and fatigue. After visiting Mada Clinics two weeks later, all medications were stopped, and the man's symptoms resolved. This case demonstrates the effects of a lack of available mental health care in Madagascar, a country with sixteen available psychiatrists for a rapidly expanding population of over 26 million people.

## 1. Introduction

**Figure 1:**
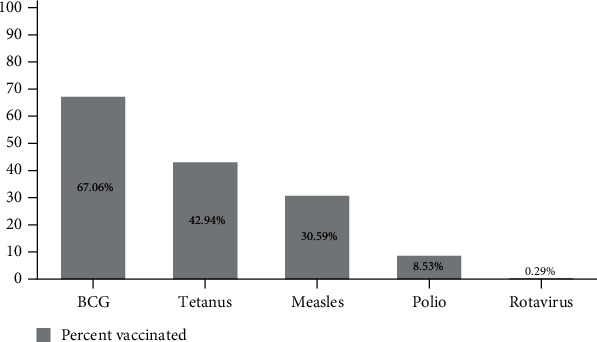
Results of vaccination survey in rural Northwestern Madagascar. 69.4% of the population in this region remains unvaccinated for measles. Vaccination rates for other major infectious diseases remain similarly low.

Madagascar is an East African Island nation with the world's seventh-lowest GDP per capita and 0.06 available psychiatrists per 100,000 population [1, 2]. This lack of psychiatric specialists not only prevents those with severe mental illness from obtaining specialized care but also correlates with a lack of mental health training in other practitioners and the absence of psychotropic medications from local pharmacies. Owing to this limited training and the difficulty in obtaining psychotropic medications, many practitioners in Madagascar treat the symptoms of psychiatric conditions with available medications, which are often better suited to somatic illnesses. In addition, vaccination rates in rural Madagascar are low, with a survey we conducted on 340 participants in the region of this case report demonstrating a measles vaccination rate of 30.6% (Figure 1).

During a 2019 measles pandemic, at least 112,000 Malagasy individuals were infected, leading to the deaths of at minimum 1200 children [3]. Loss of family members and loved ones can trigger feelings of bereavement and grief, which often manifest themselves with physical symptoms. These symptoms can include physical pain, tightness, heaviness, and shortness of breath, as well as symptoms of anxiety or panic disorders [4]. Normal grief is defined as feelings of grief that diminish within six months of loss and present with preservation of the patient's ability to function in daily life. Normal grief does not require pharmacologic treatment [5]. However, owing to the physical symptoms encountered, inexperienced practitioners may prescribe unnecessary medications that cause financial and physical harm, thereby exacerbating the adverse effects of normal grief.

We herein report a case of iatrogenic hypotension induced by improper treatment of a normal grief response. In this case, the removal of all medications was effective in reversing the patient's symptoms. To the best of our knowledge, this is the first report of iatrogenic hypotension induced by improper treatment of a normal grief response.

## 2. Case Presentation

The case was a 28-year-old male without past medical history who presented to Mada Clinics in early May 2019 with symptoms of dizziness, lightheadedness, and fatigue which made it difficult for him to maintain his rice farm. On physical exam, his blood pressure was recorded at 88/57, and upon taking a history, it was discovered that the man was taking gabapentin (900 mg/day), metoprolol (200 mg/day), and lisinopril (20 mg/day) prescribed to him by a general practice nurse for prior symptoms of intermittent chest pain, intermittent tachycardia, and widespread pain. The man lost both of his children that spring, with one child drowning in a river in February 2019, and the other child passing away in March 2019 from secondary pneumonia after contracting measles in late February 2019. He reported that in the weeks following the loss of his second child, he developed generalized full body pain along with feelings of chest pressure and a “racing heart” when he would think of his children. At that time, he was still able to carry out the normal daily work of maintaining his rice farm. The man reported that after starting the three-drug regimen of gabapentin, metoprolol, and lisinopril, his symptoms of pain and tachycardia were reduced; however, he was exhausting a significant portion of his income to afford the medications, and the side effects were making it difficult for him to complete his daily work. He subsequently presented to Mada Clinics, a nonprofit medical organization serving rural Northern Madagascar. With the exception of the aforementioned hypotension, his physical exam was otherwise normal. Suspecting that the man's original symptoms stemmed from a normal grief response, all medications were stopped, with subsequent resolution of his symptoms in the following weeks. The man did not return for his requested follow-up appointment. However, he was encountered by clinic staff while competing for his village soccer team in a regional tournament later that month where he reported resolution of all symptoms including lightheadedness, dizziness, fatigue, chest pain, generalized pain, and intermittent tachycardia.

## 3. Discussion

We have described a case of iatrogenic hypotension induced by improperly treated grief, which stemmed from the loss of life encountered during a substantial measles outbreak on the island of Madagascar in the spring of 2019. Madagascar has between 1/225^th^ to 1/500^th^ the rate of psychiatrists encountered in North America and Western Europe [1]. Exacerbating the lack of mental health services available, childhood and infant mortality remain substantial in Madagascar, leading to perhaps higher rates of grief and bereavement than those encountered in countries with greater availability of mental health services [6]. Grief can present with various physical complaints, and it is important for practitioners to understand the numerous presentations of grief to prevent improper or unnecessary treatment. Unnecessary treatment can exacerbate the burdens of grief and, particularly in developing countries, add substantial financial hardship to the bereaved patient [7]. Greater mental health training for nursing staff may have prevented the harms experienced by the patient in this case report. Of note, in the 21 years that Mada Clinics has accepted volunteer physicians from around the world, they have not once received a volunteer psychiatrist. Psychiatrists could do much by sharing their knowledge and skills with practitioners in countries with undersupplied mental health systems. More data is needed to understand the ways in which mental health complaints are currently treated in rural Madagascar.

## 4. Conclusion

Normal grief is defined as feelings of grief that diminish within 6 months of loss and present with preservation of the patient's ability to function in daily life. Normal grief does not normally require pharmacologic treatment, and such treatment can lead to unnecessary hardship for patients [5].

## Data Availability

The data used to support the findings of this study originated in written patient records kept by Mada Clinics, the details of which are included in the article. The data for the vaccine survey graph is available upon request.

## References

[1] WHO *Mental Health Atlas Country Profile Madagascar*.

[2] *GDP per capita (Current US$) - Madagascar | Data*.

[3] Nimpa M. M., Andrianirinarison J. C., Sodjinou V. D. (2020). Measles outbreak in 2018-2019, Madagascar: epidemiology and public health implications. *The Pan African Medical Journal*.

[4] Gudmundsdottir M. (2009). Embodied grief: bereaved parents' narratives of their suffering body. *Omega (Westport)*.

[5] Zisook S., Shear K. (2009). Grief and bereavement: what psychiatrists need to know. *World Psychiatry*.

[6] Sharp M., Kruse I. (2011). *Health, Nutrition, and Population in Madagascar, 2000-09*.

[7] Kruk M. E., Goldmann E., Galea S. (2009). Borrowing and selling to pay for health care in low- and middle-income countries. *Health Affairs*.

